# Evaluation of Selected Antioxidant Parameters in Ready-to-Eat Food for Infants and Young Children

**DOI:** 10.3390/nu15143160

**Published:** 2023-07-16

**Authors:** Anita Żmudzińska, Anna Puścion-Jakubik, Jolanta Soroczyńska, Katarzyna Socha

**Affiliations:** Department of Bromatology, Faculty of Pharmacy with the Division of Laboratory Medicine, Medical University of Bialystok, Mickiewicza 2D Street, 15-222 Bialystok, Poland; anna.puscion-jakubik@umb.edu.pl (A.P.-J.); jolanta.soroczynska@umb.edu.pl (J.S.); katarzyna.socha@umb.edu.pl (K.S.)

**Keywords:** antioxidant properties, processed food for infants, polyphenols, selenium, copper, zinc

## Abstract

Infants and young children have diverse dietary needs, so conducting a detailed analysis of the food they consume in terms of antioxidant activity and the content of antioxidant elements is of utmost importance. The aim of the study was to assess the antioxidant properties and the content of Cu (copper), Se (selenium), and Zn (zinc) in baby products. A total of 398 samples of ready-to-eat food consumed by children were tested. To evaluate the antioxidant activity (338 samples, without dairy), the Folin-Ciocalteu method and the 2,2-diphenyl-1-picrylhydrazyl radical scavenging test (DPPH) were employed to determine the total phenolic content (TPC). For the determination of mineral component content (398 samples), atomic absorption spectrometry (AAS) was used to analyze the levels of Cu and Zn, while inductively coupled plasma mass spectrometry (ICP-MS) was utilized for the quantification of Se. Fruit and vegetable mousses exhibited the highest average total phenolic content (TPC) and the highest percentage of free radical scavenging in the DPPH test. In terms of mineral content, the group of dairy products recorded the highest average levels of Cu and Se, while porridges contained the highest content of Zn. Notably, only organic baby food contained significantly more Zn compared to conventional food (12.2 ± 13.9 mg/kg vs. 10.7 ± 14.4 mg/kg). Ready-to-eat products designed for consumption by children provide antioxidant properties, and the presence of Zn, Cu, and Se can contribute to supporting antioxidant processes.

## 1. Introduction

Proper nutrition during infancy and early childhood is crucial for optimal development and is associated with better health outcomes later in life. The WHO recommends exclusive breastfeeding for the first six months of life, followed by the introduction of complementary foods [1]. Antioxidants derived from fruits and vegetables play a key role in the nutrition of infants and young children. A well-rounded diet that includes an adequate supply of fruit and vegetables, wholegrain products, milk, and milk products, as well as quality fats, has a positive impact on both the natural development of children and their resilience against lifestyle-related diseases. Minerals such as Zn (zinc), Cu (copper), and Se (selenium), which are involved in the antioxidant defense system, are indispensable for healthy growth and proper development during infancy.

Zn is an essential micronutrient that plays a vital role in numerous metabolic processes. It is particularly important for the proper growth and maturation of children. Zn also acts as a component of Cu–Zn superoxide dismutase (Cu/Zn SOD), thus participating in antioxidant protection [2]. Zn is classified as a type II nutrient, meaning it is essential for general metabolism as opposed to type I nutrients, which are necessary for specific bodily functions [3]. Zn deficiency in children can result in various adverse effects, including diarrhea, loss of appetite, stunted growth and development, hair loss, and increased susceptibility to infections. Excess Zn may interfere with the absorption of Cu and iron (Fe) and cause acute conditions manifested by severe nausea, vomiting, abdominal pain, and diarrhea. The tolerable upper intake level for Zn for children aged 7 to 11 months is 5 mg/day; for children aged 1–3 years, it is 7 mg/day [4]. This micronutrient is present in a wide range of foods, with animal products, shellfish, whole grains, legumes, and nuts being the richest sources. However, despite its prevalence in foods, Zn deficiencies can still occur due to the limited bioavailability of this element caused by dietary fiber and phytates [2,3]. In terms of the recommended intake, the estimated average requirement (EAR) for children aged 7 to 11 months and 1–3 years is 2.5 mg/day for Zn [5].

Cu is an essential micronutrient for the human body and is involved in many metabolic processes. It is particularly important for hematopoietic processes, the proper functioning of the nervous system, the elimination of free radicals, and immune support [6]. The sources of Cu are seafood (especially oysters), beef, offal, green leafy vegetables, legumes, cocoa, cereals, and nuts [4]. Cu can act both as an antioxidant and an oxidant, so it is important to maintain the appropriate balance between Cu and other minerals, such as Zn or manganese (Mn) [4]. Cu serves as a cofactor for various important enzymes, including cytochrome C oxidase, tyrosinase, *p*-hydroxyphenylpyrugonate hydrolase, dopamine beta-hydroxylase, lysyl oxidase, and superoxide dismutase (SOD). These enzymes play vital roles in processes necessary for growth and development [7]. It is important to maintain the homeostasis of Cu and Zn since an elevated Cu:Zn ratio can impair the antioxidant properties of a number of enzymes [4]. Both deficiency and excess of Cu in the blood can have adverse health consequences for infants and young children [8]. Cu deficiency in infants can lead to cardiovascular and immunological disorders, although such cases are relatively rare and the symptoms are not highly specific [5]. Conversely, excessive levels of Cu can lead to oxidative damage to DNA and contribute to protein and lipid oxidation [9]. As regards the recommended intake of Cu, the AI (adequate intake) for children 7–11 months is 0.3 mg/day, and the EAR for children aged 1–3 years is 0.25 mg/day [5].

Se is an essential trace element that is only required in small amounts. It is a component of oxidoreductive enzymes, including glutathione peroxidases, thioredoxin reductase, iodothyronine deiodinase, and selenophosphate synthetase 2. This is why it plays a crucial role in the immune system [3,10]. Se mediates the biosynthesis of functionally active selenoproteins, which are involved in the antioxidant defense of cells as well as in the maintenance of redox homeostasis. Selenoprotein P is a protein that plays an important role in the transport, storage, and delivery of Se in vivo. It possesses the ability to bind toxic elements and contributes to the body’s defense against oxidative stress [10]. The primary dietary sources of Se are plant products, including vegetables, whole grains, meat, seafood, and fish. However, it is important to note that the actual content of this element strictly depends on the content of Se present in the soil where animals are raised or plants are cultivated [3,10]. Se deficiency is rare and usually occurs in countries with low soil concentrations. It can also be seen in cases of total parenteral nutrition and in infants fed cow’s milk instead of breast milk [11]. An insufficient level of Se can contribute to a weakened immune system, hypothyroidism, and heart disease [3]. Deficiency can cause cardiomyopathies in children, although it is mainly observed in China [11]. Excess Se can also be toxic and manifest in disorders of the musculoskeletal system, and the tolerable upper intake level for Se is 60 µg/day [11]. According to the recommendations, the AI for children aged 7–11 months is 20 µg/day and the EAR of Se for children aged 1–3 years is 17 µg/day [5].

Many methods are used to assess the antioxidant properties of food. Among the most popular ones are the assessment of the total phenolic content (TPC) using the Folin-Ciocalteu reagent (F-C) and the radical scavenging test using 2,2-Diphenyl-1-picrylhydrazyl (DPPH) [12]. The basic mechanism of action of F-C involves oxidation and reduction processes, where the oxidation of phenols with the F-C reagent produces a color reaction [13]. The DPPH test is used to determine the ability of compounds to scavenge free radicals [12]. Other methods described in the literature include the determination of the reduction of iron ions (FRAP), the assessment of the ability to absorb oxygen radicals (ORAC), and the method using a reagent (ABTS) [14].

Ready-to-eat processed foods for infants and young children are distinct from non-specific processed foods for adults due to the stringent compositional and health safety requirements outlined in the Commission Directive 2006/125/EC of 5 December 2006, which specifically addresses processed cereal-based foods and baby foods for infants and young children [15]. The DONALD study showed that 20% of infants and young children consumed home-cooked meals, while 60% were fed processed, ready-to-eat products. The remaining 20% had a combination of homemade and ready-to-eat foods designed for children [16]. Additionally, the CHOP study observed that over 95% of children aged 9–12 months consumed at least one ready-to-eat product [17]. Given the high prevalence of ready-made, processed products for children, it is important that this kind of food maintain high quality standards and appropriate composition. While the antioxidant properties and antioxidant vitamin content of foods have been extensively studied in the literature, there is a dearth of scientific reports specifically focusing on finished products intended for children.

Previous research on ready-to-eat products for children has focused on the assessment of food contaminants, while there are few studies assessing the antioxidant properties of foods. A number of studies are proposed in the literature, however, the number of samples and variety of products for children are limited (from *n* = 7 to *n* = 60 [18,19,20,21,22,23,24,25,26,27,28]). According to the latest knowledge, the current scientific reports focus on the analysis of children’s dinners and fruit and vegetable mousses [18,19,20,21,22,23,24,25,26,27,28], and there is little or no data describing the antioxidant properties in children’s drinks, porridges, snacks, and dairy products.

The objective of the study was to evaluate the antioxidant properties of ready-to-eat products for children aged 0.5–3 years. The assessment includes analyzing the concentrations of Zn, Cu, and Se, as well as the antioxidant activity determined by the content of phenolic compounds using the F-C method and the DPPH test. This study included samples from European and non-European producers to take into account the diversity of this type of food.

## 2. Materials and Methods

### 2.1. Sample Collection and Preparation

**Figure 1:**
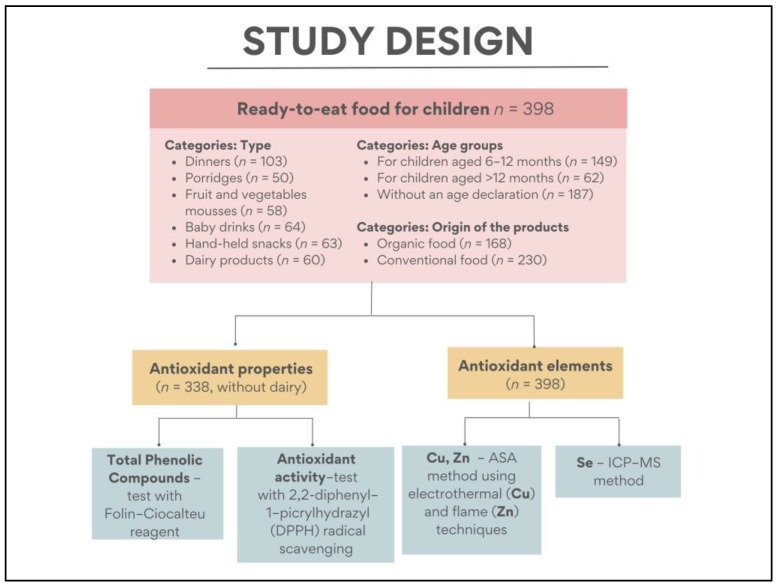
Research project on the antioxidant properties of food for children. Cu—copper, n—number of samples, Se—selenium, Zn—zinc.

The research material consisted of 398 products intended for consumption by children. These products included baby dinners (*n* = 103), porridges (*n* = 50), fruit and vegetable mousses (*n* = 58), baby drinks (*n* = 64), hand-held snacks (*n* = 63), and dairy products (*n* = 60). Within each category, a large variety of products was collected to ensure representative groups for analysis. The samples were selected to reflect the assortment of products available in the Polish market. The samples were purchased from various sources: brick-and-mortar stores (discount stores, hypermarkets, and drugstores with children’s food in Białystok, Poland) and online stores. The analyzed products were sourced from leading manufacturers of ready-to-eat products for infants and young children, including Nutricia, Nestle, Humana, Hipp, Holle, and Helpa. While the majority of the tested samples are suitable for consumption by children from 6 months of age, certain products (e.g., biscuits, chocolate bars, or cookies) are recommended for consumption after 12 months of age. The study design is shown in Figure 1.

The procedure for sample preparation involved homogenizing the samples in a mill or grinding them in a mortar. The ground samples were then weighed, with a sample weight ranging from 0.25 to 0.35 g, and measured with an accuracy of 0.001 g. These weighed samples were placed in mineralization vessels made of polytetrafluoroethylene. Next, 4 mL of concentrated 69% HNO_3_ (Tracepur, Merck, Darmstadt, Germany) was added to each sample. Microwave digestion was conducted in a closed-loop system (Berghof, Speedwave, Eningen, Germany). The mineralization process consisted of four phases. The first phase lasted 10 min at 170 °C, 20 atm pressure, and 90% power; the second phase lasted 10 min at 190 °C, 30 atm pressure, and 90% power; and the third phase lasted 40 min at 210 °C, 40 atm pressure, and 90% power. The fourth phase involved cooling time and lasted 18 min, at 50 °C, 40 atm pressure, and 0% power. After mineralization, the samples were quantitatively transferred to polypropylene vessels and diluted.

### 2.2. Determination of Mineral Components

The mineral concentrations in the samples were analyzed using different analytical techniques. Flame Atomic Absorption Spectrometry (AAS) with Zeeman background correction was employed to analyze Zn. The measurements were performed at a wavelength of 324.8 nm using the Z-2000 instrument from Hitachi, Tokyo, Japan. For Cu analysis, electrothermal Atomic Absorption Spectrometry with Zeeman background correction was utilized. The measurements were conducted at a wavelength of 213.9 nm. Se analysis was conducted using Inductively Coupled Plasma Mass Spectrometry with kinetic energy discrimination (KED). The instrument used was the NexION 300D from PerkinElmer, Waltham, MA, USA. The detection limits (LOD) for Zn, Cu, and Se were 0.018 mg/kg, 0.39 µg/kg, and 0.01 µg/kg, respectively. The LOD was calculated as three times the standard deviation from the mean value of the blank sample. The limit of quantification (LOQ) value was calculated as three times the LOD and LOQ values for Zn, Cu, and Se which were 0.054 mg/kg, 1.17 µg/kg, 0.03 µg/kg, respectively.

In order to evaluate the accuracy and precision of the study, certified reference materials (CRMs) were used. For the analysis of baby dinners, the material used was Simulated Diet D obtained from the Swedish National Food Administration (Livsmedelsverket, Uppsala, Sweden). Porridges and snacks were tested using corn flour INCT-CF-3 obtained from the Institute of Nuclear Chemistry and Technology (Warsaw, Poland). The analysis of dairy products utilized skim milk powder CRM 063R provided by the Community Bureau of Reference (BCR). A total of six independent samples were analyzed for each CRM.

The concentrations of mineral components were also expressed as a percentage of the Estimated Average Requirement (EAR) coverage. This calculation was based on standard portions consumed by children, as declared by the manufacturer [28]. The Cu:Zn molar ratio was calculated for all samples using Microsoft Excel software.

### 2.3. Determination of Antioxidant Properties

The TPC was determined using the Folin-Ciocalteu reagent [29]. To create the calibration curve, a solution of gallic acid (GAE) with a concentration of 2 g/L in distilled water was prepared. The samples were homogenized, weighed (1 g ± 0.001 g), dissolved in 10 mL of distilled water, and then centrifuged for 5 min at 2000 rotations per min (rpm). Next, 0.25 mL of the supernatant was taken and mixed with 1.25 mL of 0.2 N Folin-Ciocalteu reagent. The mixture was allowed to react for 5 min. The absorbance of the resulting solution was measured at 760 nm against water. The concentration was expressed as mg of equivalent gallic acid/100 g of the product. The reported result represents the average of three measurements.

The antioxidant activity of the samples was also determined using the radical scavenging test with DPPH [30]. The samples were homogenized, and 5 g ± 0.001 g were measured. Next, 10 mL of 80% ethanol was added to the samples, and the mixture was shaken for 15 min. A DPPH solution was prepared by dissolving 10 mg of DPPH in 100 mL of 80% ethanol. Subsequently, the samples were centrifuged for 3 min, at 5000 rpm. After some time, 2 mL of the supernatant was taken and mixed with 2 mL of DPPH solution. As a control, 2 mL of the DPPH mixture was mixed with 2 mL of 80% ethanol. The samples were incubated for 30 min at room temperature, protected from light. The absorbance of the resulting solutions was measured at 517 nm against 80% ethanol with a spectrophotometer U-2001 (Hitachi, Tokyo, Japan). The percentage of free radical scavenging was calculated using the following equation:

DPPH inhibition [%] = [(A_control_ − A_sample_)/A_control_] × 100%,

where A_control_ is the absorbance of the control reaction and A_sample_ is the absorbance of the analyzed sample.

### 2.4. Statistical Analysis

The obtained data were analyzed using the Statistica software (TIBCO Software Inc., Palo Alto, CA, USA). The Shapiro–Wilk test was performed, and the distribution was found to be non-normal. Non-parametric Mann–Whitney U tests and the Kruskal–Wallis ANOVA test were used to compare the values of the tested parameters in various product groups. Spearman’s rank correlation was used to check the relationship between the analyzed parameters tested in all product subgroups. The results were summarized using the median and quartiles, however, to compare the results of our own research with those of other authors, the tables contain the mean, along with the standard deviation, as well as the maximum and minimum values. Significant difference values were assumed at *p* < 0.05, *p* < 0.01, and *p* < 0.001.

## 3. Results

### 3.1. Evaluation of Antioxidant Properties: DPPH, TPC

The contents of TPC, DPPH, Cu, Se, Zn, and the Cu: Zn ratio, along with the corresponding statistical significance, are presented in Table 1.

In the analyzed assortment, the median TPC was found to be 37.8 mg GAE/100 g (Q1–Q2 Q1—quartile 1, Q2—quartile 2: 12.4–82). The group of products based on fruit and vegetable mousses had the highest median TPC, recording 111.8 mg GAE/100 g (55.9–162.4), and the highest value within this subgroup was 114.2 (65.8–167.4) mg GAE/100 g (Supplementary Materials, Table S3). On the other hand, the group of dinners for children had the lowest median TPC of 25.8 mg GAE/100 g (14.2–37.0), with fish-based dinners having the lowest TPC of 17.3 mg GAE/100 g (7.6–33.3) (Table S1). Among the products for children, those with the highest TPC were found to be freeze-dried fruits consisting of blackcurrant, pineapple, cherry, and strawberries (525.4 mg GAE/100 g), freeze-dried fruits based on strawberries, raspberries, blueberries, and apples (507.2 mg GAE/100 g), and apple chips (312.0 mg GAE/100 g). It is worth noting that TPC was not detected in nine products.

Baby products exhibited a free radical scavenging capacity of 71.4% (48.0–86.8). The group of fruit and vegetable mousses had the highest median obtained in the DPPH test, with a value of 95.3% (91.0–99.6). Among the subgroups, fruit mousses had the highest free radical scavenging capacity of 95.9% (87.2–97.4) (Table S3). On the other hand, the group of drinks for children showed the lowest percentage of free radical scavenging in the DPPH test, recording 34.8% (2.8–52.5), with fruit juices exhibiting the lowest capacity within this subgroup, namely 33.7% (4.4–56.6) (Table S4). Summer fruit salad containing pear, apple, mirabelles, and apricots (99.6%), applesauce (99.5%), and banana, mango, and coconut milk mousse (89.6%) had the highest free radical scavenging capacities.

### 3.2. Evaluation of Mineral Components: Cu, Se, Zn

In products for children, the median concentration of Cu was 7.8 mg/kg (4.4–12.1). The group of dairy products exhibited the highest median Cu content: 18.2 mg/kg (14.0–22.9). Among the subgroups, yogurts showed the highest median Cu value: 22.1 mg/kg (20.4–26.6) (Table S6). The lowest Cu content was recorded in baby drinks, with 2.1 mg/kg (1.8–4.6), and within the subgroups, fruit juices had the lowest Cu content: 1.9 mg/kg (1.7–4.0) (Table S4). Among the products for children, those with the highest content of Cu were: organic cereal and fruit bars based on oat flakes, bananas, and apples (90.7 mg/kg); organic fruit and cereal bars based on oat flakes, bananas, apples, and grapes (90.3 mg/kg); and yogurt with chocolate chips (51.7 mg/kg).

The median concentration of Se in products for children was 65.0 µg/kg (30.1–100.2). The group of dairy products exhibited the highest median Se content of 134.9 µg/kg (113.9–195.5). Among the subgroups, yellow cheese showed the highest median Se content—164.2 µg/kg (120.2–214.5) (Table S6). The lowest median Se content was observed in dinners, with 24.5 µg/kg (16.4–32.2), while within the subgroups, poultry-based dinners had the highest content of this element—16.6 µg/kg (13.2–25.5) (Table S1). Among the products for children, those with the highest Se content were organic oatmeal (686.6 µg/kg), tea based on lemon balm, linden, and marjoram (567.2 µg/kg), and lemonade based on apple, strawberry, rosehip, and mint (485.2 µg/kg).

The median Zn content in products intended for infants and young children was 7.8 mg/kg (4.4–12.1). Among the different groups, children’s porridges were found to have the highest content of Zn at 27.4 mg/kg (18.8–37.1). Within the subgroups, milk porridges had the highest content, with a median of 34.4 mg/kg (18.5–44.0) (Table S2). The lowest median of Zn was found in baby drinks at 1.3 mg/kg (0.7–2.6), while among the subgroups, fruit juices had the lowest Zn content at 1.0 mg/kg (0.7–1.9) (Table S4). Products for children with the highest content of Zn were: dairy-free buckwheat porridge (93.3 mg/kg), milk banana porridge made of wholegrain cereals (79.7 mg/kg), and a bio cereal bar based on oat flakes, apples, and bananas (69.4 mg/kg).

The median molar ratio of Cu:Zn in baby products was 1.7 (0.6–3.3). Fruit and vegetable mousses exhibited the highest Cu:Zn molar ratio—4.8 (3.1–7.8), followed by dairy products—3.4 (0.6–5.2). The lowest Cu:Zn molar ratio was found in children’s porridges at 0.1 (0.0–0.2) and snacks at 0.8 (0.5–1.3).

### 3.3. EAR of Cu, Se, and Zn

The analyses were conducted to determine the extent to which a portion of products for children covered the average requirement for Cu, Zn, and Se. The results revealed that the average portion of baby products covered 232.0% of the EAR for Cu, 19.2% for Zn, and 24.3% for Se. Among the product groups, fruit and vegetable mousses showed the highest percentage of Cu and Se coverage, with 492.9% for Cu and 52.8% for Se. In the case of Zn, hand-held snacks had the highest coverage (54.5%). The average percentages of EAR coverage of trace elements in baby products are presented in Table 2.

### 3.4. Evaluation of the Content of Cu, Se, Zn, and DPPH, TPC in Baby Products Taking into Account the Intended Use of Products for the Age Groups

Ready-to-eat products for children were analyzed in terms of their intended use and divided into age categories: for infants 6–12 months, for children between 1 and 3 years, as well as products without an age declaration. Table 3 presents the characteristics of the age groups, taking into account the mean, median, and other statistical parameters. The age-declared group included dinners, porridges, and processed vegetables and fruits. Other groups (dairy products, snacks, and drinks) were categorized without age values. The highest median values for TPC were recorded in the group of products for children without an age declaration, at 50.8 mg GAE/100 g (15.9–99.1), and in products for children aged 6–12 months, at 42.6 mg GAE/100 g (21.1–98.7). Significantly lower TPC content was found in the group of products for children between 1 and 3 years (24.0 mg GAE/100 g (12.2–38.2). Regarding the DPPH test, statistically higher values were recorded in the group of products intended for children aged 6–12 months, with a value of 79.9% (56.8–93.3,) and in products for children between 1 and 3 years, with a value of 77.8% (67.8–88.5), compared to products without an age declaration, which had a value of 53.4% (30.1–76.9). The highest median Cu values were found in products without an age declaration: 10.7 mg/kg (2.2–17.6). The concentrations were significantly lower in products for children 6–12 months: 6.9 mg/kg (4.1–9.2) and between 1 and 3 years: 6.9 mg/kg (5.3–8.6). The group of products without an age declaration also recorded the highest median Se concentration, which was 93.0 µg/kg (68.0–127.7). Products for children aged 6–12 months had a median Se concentration of 39.8 µg/kg (22.5–71.6), and products for children between 1 and 3 years had the lowest Se concentration, measuring 26.1 µg/kg (17.0–41.7). As regards Zn, products for children aged 6–12 months had a concentration of 3.7 mg/kg (2.2–12.0), while products for children between 1 and 3 years contained 5.8 mg/kg (2.8–9.6). Products without an age declaration had a similar concentration of 5.5 mg/kg (2.2–17.6), and these results were not statistically significant. Medians, quartiles, and other statistical parameters of TPC, DPPH, Cu, Zn, and Se, taking into account the intended use of products for age groups, are presented in Table 3.

### 3.5. Evaluation of the Content of Cu, Se, Zn, and DPPH, TPC in Baby Products Taking into Account Food Origin

Products intended for consumption by children were also analyzed, considering the origin of the raw materials. The analysis distinguished between the organic food group, consisting of 168 samples (including 50 dinners, 17 porridges, 23 fruit and vegetable mousses, 28 drinks, and 50 snacks), and the conventional food group, consisting of 230 samples (including 53 dinners, 33 porridges, 35 fruit and vegetable mousses, 36 drinks, 13 snacks, and 60 dairy products). The results are presented in Table 4. It was found that organic food had higher medians in the DPPH test, with a median of 72.7% (48.6–85.5), compared to conventional food, with a DPPH of 69.5% (43.8–89.5). Similarly, organic food had higher median concentrations of Se: 69.5 µg/kg (28.8–106) and Zn: 6.8 mg/kg (2.8–16.3) compared to conventional food, with Se: 64.4 µg/kg (30.5–91.8) and Zn: 4.3 mg/kg (2.0–12.4). On the other hand, conventional food showed higher medians of TPC, with a median of 44.6 mg GAE/100 g (15.8–96.8), and Cu, with a median of 51.7 mg/kg (4.2–13.2), compared to organic food, with a TPC median of 34.3 mg GAE/100 g (16.8–78.2) and a Cu median of 7.2 mg/kg (5.2–10.9). Statistically significant differences were observed only in the concentration of Zn. Medians, quartiles, and other statistical parameters of TPC, DPPH, Cu, Zn, and Se, taking into account the origin of the products, are presented in Table 4.

### 3.6. Correlations

The analysis of correlations between the evaluated parameters showed a high relationship between Se and Zn in the group of drinks for children (r = 0.56, *p* < 0.001). Among other parameters, the average correlation between Se and Zn in baby dairy products (r = 0.45, *p* < 0.001), Se and Cu in baby drinks (r = 0.38, *p* < 0.005), Cu and DPPH in porridges (r = 0.38, *p* < 0.001), Zn and Cu in dinners (r = 0.35, *p* < 0.001), Cu and DPPH in total products (r = 0.38, *p* < 0.001) should be emphasized. Correlations between individual parameters are presented in Table 5.

## 4. Discussion

Food for children should ideally possess antioxidant properties, which is why the study aimed to determine the antioxidant activity of the analyzed samples. In our study, the median TPC was found to be 37.8 mg GAE/100 g. Baby products exhibited a free radical scavenging capacity of 71.4%. In products for children, the median concentrations of Cu, Se, and Zn were respectively 7.8 mg/kg, 65.0 µg/kg, and 7.8 mg/kg.

In research conducted by Usal et al. (2020), the TPC value in fruit and vegetable-based baby food jars was reported to be 1310.9 ± 174.6 mg GAE/100 g [18]. On the other hand, Carbonell-Capella et al. (2014), who investigated the antioxidant properties of 23 fruit preserves, revealed that the mousse based on apple, pear, peach, and apricot, which had undergone the cooking process, had the highest average content of phenols (234.2 mg GAE/100 g). In our study, TPC values were also the highest in processed fruit and vegetables, although they were much lower compared to those reported by other authors (112 ± 65.37 mg GAE/100 g). Additionally, our study found that freeze-dried fruit, specifically currants, pineapple, cherry, and strawberry, had the highest content of phenolic compounds, measuring 525.43 mg GAE/100 g) [19].

The ability to capture free radicals in baby foods has not yet been measured, but vegetables and fruits have the highest percentage of DPPH radical scavenging [31]. In our study, fruit, and vegetable mousses were also found to have the highest DPPH value (100.0 ± 17.6%). In a study by Deng et al. (2019), the scavenging rate of DPPH radicals in juices was 77% [32]. In contrast, our study found that juices for children had a much lower ability to capture free radicals (24.7 ± 50.1%). Furthermore, it has been observed that the storage of juices can reduce the percentage of scavenging free radicals [33]. Baby products are pasteurized and have a long shelf life, which may explain the lower percentages in the DPPH test for baby products. Szajdek et al. (2007) found that apple-currant mousse had the highest free radical quenching activity [20]. Our study yielded similar results. The products with the highest percentage of scavenging free radicals were fruit salads containing apples and apple mousse.

Vegetable and fruit-based foods are the greatest sources of antioxidants, which is why ready-to-eat foods for children have antioxidant properties. Vegetable and fruit mousses are the least processed products, which is why they have the highest antioxidant activity. It is worth considering recommending fruit and vegetable mousses to children as an alternative to the consumption of raw fruit and vegetables because these products are some of the best sources of antioxidants.

Cu, Zn, and Se are essential micronutrients involved in many metabolic processes. The content of these elements in the tested samples showed variation. In a Spanish study by Mir-Marques et al. (2015), the percentage of AI (adequate intake) coverage in meat dinners, fish dinners, vegetable jars, and fruit jars was examined. The research found that the dietary contribution of Cu in these complementary products for children was, on average, 42%, 30%, 46%, and 64% of the AI, respectively [21]. In our study, we estimated the percentage of EAR, and in most cases, the demand was covered. However, porridge (35.1 ± 20.4%) and baby drinks (58 ± 35.4%) had insufficient EAR coverage for Cu. On the other hand, children’s dinners had a notably high coverage of 167.1 ± 89.1%, and children’s dairy showed an even higher coverage of 196.4 ± 184.1%. Interestingly, fruit and vegetable mousses demonstrated a significantly higher percentage of Cu coverage (492.9 ± 300%), as did hand-held snacks (465.2 ± 477.3%), which is a concerning finding.

In comparison to our study, other publications have reported much lower Cu content in children’s products. For example, Zand et al. (2022) evaluated the concentration of essential and trace elements in commercial food and found that the average Cu content in meat dinners was 0.5 mg/kg, while in vegetarian dinners, the content of this element was below the limit of detection (LOD) [34]. Škrbić et al. (2017) examined the content of essential elements, including Cu, in food from the Spanish and Serbian markets. In products for children on the Serbian market, the average Cu content in meat lunches was found to be 0.46 mg/kg, in vegetable jars—0.28 mg/kg, in fruit porridges—0.75 mg/kg, in corn and rice porridges—0.56 mg/kg, and in fruit yogurts—0.44 mg/kg. On the Spanish baby food market, the average Cu content in poultry-based dinners was 0.11 mg/kg, and in fish-based dinners, it was 0.16 mg/kg [23].

In a Polish study by Marzec et al. (2005), the average concentration of Cu in baby drinks was reported to be 0.31 mg/kg, and in dinners, it was 0.42 mg/kg [9]. In our study, higher Cu concentrations were obtained. Meat dinners contained from 6 ± 3.3 mg/kg to 7.7 ± 2.9 mg/kg Cu; vegetarian dinners had 7.4 ± 2.5 mg/kg; fruit porridges had 2.2 ± 1.2 mg/kg; yogurt had 23.8 ± 7.6 mg/kg; and baby drinks had 3.4 ± 2.1 mg/kg.

In a study by Khamoni et al. (2017), the authors examined the content of Cu in baby foods and obtained similar results. The average concentration of Cu in products intended for children over 7 months old was 11.8 ± 0.83 mg/kg, and in food for children over 10 months old, it was 14.3 ± 0.9 mg/kg [24].

In most cases, ready-to-eat foods for children can be a source of Cu. Cereals and drinks for children can be problematic in providing the right amount of Cu. On the other hand, mousses and snacks for children provide very large amounts of Cu (>400% of EAR) with low amounts of Zn (<50% of EAR), which may reduce their antioxidant properties. Summing up our own and other authors’ results, Cu is present in products for children in greater amounts, which is why an excess of Cu may be problematic, especially with an insufficient content of Zn.

In the UK study, Se content in baby products was similar to our results. In products intended for children aged 7 and more months, the Se content was 8.8 ± 2, while in products for children over 10 months, it was 10.3 ± 3 µg/kg [24].

Ruiz-de-Cenzano et al. (2017) studied Se content in commercial baby food. The concentrations of Se in children’s products ranged from 5.4 to 109 µg/kg. In our research, the Se content in products for children is very diverse (<LOD–383.6 µg/kg). The highest values in the Ruiz-de-Cenzano study were recorded in fish-based dinners (from 44 to 109 µg/kg) and meat-based dinners (from 15 to 36 µg/kg). The lowest Se concentrations were found in products based on vegetables and fruits (below 10 µg/kg) [25]. In our study, fruit, and vegetable mousses contained an average of 80.4 ± 37 µg/kg, and the lowest Se values were recorded in children’s lunches, with levels below 26.7 ± 14.3 µg/kg. The discrepancies in the results may be attributed to variations in Se content in the soil and animal feed. It is also worth emphasizing that children’s products covered 24.3 ± 22.9% of the EAR for Se.

Among all ready-to-eat products for children, dairy is the best source of Se. Therefore, special attention should be paid to the adequate supply of Se in children on a dairy-free diet.

In a study by Butte et al. (2010), it was observed that the intake of Zn in some children was insufficient, while in another group of children up to 24 months who were supplementing with Zn, the intake reached the recommended level [35]. Other researchers estimated that food for infants and young children covered approximately 16% of the demand for Zn [36]. Zand et al. (2011) demonstrated that the average Zn content of meat-based dinners was 5.4 mg/kg, while vegetable-based dinners had an average of 3.4 mg/kg [34]. Our study yielded similar results: meat dinners for children contained, on average, 3.7 ± 2.3 mg/kg to 6.4 ± 1.9 mg/kg of Zn, while vegetarian dinners contained 7.8 ± 9.6 mg/kg of Zn. Zand et al. also analyzed Zn content in poultry-based and fish-based dinners, and Zn values were reported as 2 mg/kg for both types of dinners. [22]. In our study, poultry-based dinners were found to contain 5.4 ± 4 mg/kg of Zn, while fish-based dinners had 3.7 ± 2.3 mg/kg of Zn. Similar values were reported by Kiani et al. (2022), where the average concentration of Zu in children’s products was 1.8 ± 0.6 mg/kg [26].

In a study by Vallinoto et al. (2022), Zn content in children’s products ranged from 0.45 ± 0.02 mg/kg (apple-based product) to 10.6 ± 0.5 mg/kg (meat-based lunch with vegetables). This group of researchers also observed that products containing milk in the composition had a higher concentration of Zn, which is consistent with our results because Zn is considered an important source [27].

Analogous results were also reported in the study by Khamoni et al. (2017), where products for children over 7 months contained 8.8 ± 0.2 mg/kg and products for children over 10 months contained 10.5 ± 0.5 mg/kg. In this study, the Cu:Zn molar ratio was also assessed, and in the group of products over 7 months, it was 3.0, while in the group of products over 10 months, it was 8.0. In our study, the molar ratio was similar at 2.8 [24].

In Marzec et al. (2005), the average concentration of Zn in children’s drinks and dinners was 2.6 mg/kg (our study: 4.2 ± 7.8 mg/kg) and 2.98 mg/kg (our study: 5.3 ± 5.1 mg/kg), respectively [9]. An analysis of the percentage of EAR nutritional coverage by Mir-Marques et al. (2015) found that Zn accounted for 35% in meat-based dinners, 12% in fish-based dinners, 17% in vegetable jars, and 7% in fruit jars [21]. Our study yielded similar results. Baby dinners covered 12.9 ± 14.6% of the EAR for Zn, while fruit and vegetable mousses covered 9.7 ± 4.6% of the demand for the element.

It is also worth noting the high variability of the Cu:Zn ratio. So far, no recommended Cu/Zn ratio has been established for products intended for infants and young children. However, studies have shown that low concentrations of Zn and increased concentrations of Cu can diminish the antioxidant activity of many enzymes [4]. A high Cu:Zn ratio in the blood may indicate inflammation and a high risk of Zn deficiency in children with chronic diseases [37]. In our study, the highest Cu:Zn molar ratio was observed in fruit and vegetable mousses (6.5 ± 5.4), while the lowest was in children’s porridges (0.2 ± 0.1).

Baby food is one of the most frequently purchased organic products, and, according to the ESKiMo II study, as many as 63% of children consume organic food [38]. However, there is no confirmed evidence that organic food is significantly more nutritious than conventional food [39]. Our study shows that organic food for children only contains a significantly higher amount of Zn compared to conventional food. Currently, there is insufficient scientific evidence to conclude that organic food has a significantly greater health value [40], and the American Academy of Pediatrics does not advocate choosing organic food over conventional food [41].

It is important to note that we found variations in the antioxidant activity and the levels of Cu, Se, and Zn in products for infants aged 6–12 months, those for children over 12 months, and products without an age declaration. Products intended for infants aged 6–12 months exhibited significantly higher DPPH capture percentages and higher concentrations of Cu and Se compared to products for children without an age declaration. Products designed for infants aged 6–12 months had higher values of TPC and Se when compared to products without an age specification. The lack of consistency in the composition of children’s products across different age groups poses a limitation in conducting a comprehensive assessment of age categories. Consequently, we cannot ascertain the exact reason why products meant for infants aged 6–12 months may have a higher antioxidant value.

The strength of this study is that it includes a wide variety of complementary products for children and incorporates a substantial number of samples, providing a comprehensive analysis of their antioxidant properties. This research represents a pioneering effort in investigating the antioxidant properties of such a diverse range of ready-to-eat products specifically tailored for young children. Notably, our study is the largest of its kind, assessing the content of Cu, Se, Zn, and antioxidant properties in products intended for children on the Polish market. The limitation of the study is the lack of consistency in the composition of children’s products; therefore, the reasons for the above results cannot be identified comprehensively.

## 5. Conclusions

Ready-made products for children possess antioxidant properties and high levels of Cu, Se, and Zn; therefore, they can support antioxidant processes. It is crucial to ensure that the nutrition of infants and young children aged 0.5–3 years is diverse to minimize the risk of inadequate antioxidant intake. Given the high molar ratio of Cu:Zn, it is worth paying attention to the appropriate consumption of Zn-rich products in children’s diets, especially in the case of high consumption of fruit and vegetable mousses and low consumption of porridges.

## Figures and Tables

**Table 1:** The content of TPC, DPPH, Cu, Se, Zn, and the Cu:Zn ratio in tested products.

Type of Product (Sign)		TPC (mg GAE/100 g of Fresh Weight)	DPPH (% Free Radical Scavenging)	Cu (mg/kg of Fresh Weight)	Se (µg/kg of Fresh Weight)	Zn (mg/kg of Fresh Weight)	Cu:Zn Molar Ratio
Dinners (D) *n* = 103	M ± SD	29.1 ± 23.3	59.3 ± 29.5	7.3 ± 2.5	26.7 ± 14.3	5.3 ± 5.1	2.0 ± 1.2
	Min-Max	(0–181.6)	(0–96.9)	(1.9–15.6)	(5.3–76.4)	(0.8–46.3)	(0.1–6.4)
	Me	25.8 (14.2–37.0)	69 (50.8–79.2)	7 (5.7–8.5)	24.5 (16.4–32.2)	4.0 (2.8–6.6)	1.7 (1.2–2.4)
	Q1–Q3	*** FV, BD	*** FV, BD	*** P, FV, BD, S, DP	*** FV, BD, S, DP	*** P, FV, BD, S, D	** S, *** P, FV
Porridges (P) *n* = 50	M ± SD	44.9 ± 45.9	68.5 ± 27.0	3.8 ± 2.2	55.6 ± 95.5	30.4 ± 16.5	0.2 ± 0.1
	Min–Max	(3.6–206.0)	(0–97.0)	(0.5–9.2)	(5.2–686.6)	(3.7–93.3)	(0–0.4)
	Me	33.5 (12.5–69.2)	77.9 (59.1–86.8)	3.4 (1.8–5.4)	33.4 (22.3–65.1)	27.4 (18.8–37.1)	0.1 (0–0.2)
	Q1–Q3	*** FV	*** FV, BD	*** D, FV, S, DP	*** FV, BD, S, DP	*** D, FV, BD	*** D, FV, BD, S
Fruit and Vegetable–mousses (FV) *n* = 58	M ± SD	112.0 ± 65.4	100.0 ± 17.6	11.0 ± 6.7	80.4 ± 37.0	2.2 ± 1	6.5 ± 5.4
	Min–Max	(6.5–250.8)	(0–99.6)	(2.2–39.5)	(<LOQ-175.7)	(0.6–4.8)	(0.8–30.9)
	Me	111.8 (55.9–162.4)	95.3 (91.0–99.6)	9.3 (7.3–14.2)	79.3 (53.3–102.5)	2.2 (1.3–2.9)	4.8 (3.1–7.8)
	Q1–Q3	*** D, P, S	*** D, P, BD, S	* D, DP, *** P, BD	*** D, P	*** D, P, S, DP	** DP, *** D, P, BD, S
Baby drinks (BD) *n* = 64	M ± SD	75.9 ± 55.8	22.8 ± 46.0	3.4 ± 2.1	116.7 ± 110.0	4.2 ± 7.8	3.9 ± 8.5
	Min–Max	(0–242.0)	(0–87.4)	(1.3–8.7)	(<LOQ-567.2)	(0.1–39.0)	(0.2–62.5)
	Me	74.7 (33.0–105.4)	34.8 (2.8–52.5)	2.1 (1.8–4.6)	87.7 (56.7–111)	1.3 (0.7–2.6)	1.9 (0.9–3.4)
	Q1–Q3	*** D	*** D, P, FV, S	*** D, FV, S, DP	*** D, P	*** D, P, S	*** P, FV, S
Hand-held snacks (S) *n* = 63	M ± SD	68.1 ± 103.1	66.6 ± 85.6	14.3 ± 14.7	76.6 ± 21.6	16.7 ± 10.7	1.1 ± 0.9
	Min–Max	(0–525.4)	(0–91.7)	(2.9–90.7)	(24.1–122.2)	(3.9–69.4)	(0.2–6.2)
	Me	29.3 (12.4–82.0)	77.0 (56.1–85.6)	11.5 (8.7–13.9)	78.9 (65.4–91.2)	14.4 (10.4–20)	0.8 (0.5–1.3)
	Q1–Q3	*** FV	*** FV, BD	* FV *** D, P, BD	*** D, P	*** D, FV, BD	** D *** P, FV, BD, DP
Dairy products (DP) *n* = 60	M ± SD	-	-	19.4 ± 7.5	163.2 ± 84.8	16.3 ± 18.2	3.5 ± 2.8
	Min–Max			(9.1–51.7)	(47.5–454.7)	(2–63.7)	(0.3–11.2)
	Me			18.2 (14–22.9)	134.9 (113.9–195.5)	5.8 (4.3–38.7)	3.4 (0.6–5.2)
	Q1–Q3			* FV *** D, P, BD	*** D, P	*** D, FV, BD	** FV *** P, S
TOTAL *n* = 398	M ± SD	61.8 ± 67.7	60.5 ± 38.0	9.7 ± 9.1	81.1 ± 80.7	11.3 ± 15.0	2.8 ± 4.6
	Min–Max	(0–525.4)	(0–99.6)	(0.5–90.7)	(<LOQ-686.6)	(0.1–93.3)	(0–62.5)
	Me Q1–Q3	37.8 (16.6–86.7)	71.4 (48–86.8)	7.8 (4.4–12.1)	65.0 (30.1–100.2)	7.8 (4.4–12.1)	(0.6–3.3)

* *p* < 0.05, ** *p* < 0.01, *** *p* < 0.001. BD—baby drinks, Cu—copper, D—dinners, DP—dairy products, DPPH—2,2-diphenyl-1-picrylhydrazyl radical scavenging test, FV—fruits and vegetable mousses, GAE—gallic acid, LOQ—limit of quantification, M—mean, Max—maximum, Me—median, Min—minimum, *n*—number of samples, P—porridges, Q1—quartile 1, Q3—quartile 3, S—snacks “for the hand”, SD—standard deviation, Se—selenium, TPC—total phenolic content, Zn—zinc.

**Table 2:** Coverage in % of the Estimated Average Requirement (EAR) for Cu, Zn, and Se in baby products.

Type of Product	Elements	Dinners	Porridge	Fruit and Vegetables Mousses	Baby Drinks	Hand-Held Snacks	Dairy Products	Total
% EAR	Cu	167.1 ± 89.1	35.1 ± 20.4	492.9 ± 300.0	58 ± 35.4	465.2 ± 477.3	196.4 ± 184.1	232.0 ± 291.9
	Zn	12.9 ± 14.6	28.2 ± 15.3	9.7 ± 4.6	7.3 ± 13.4	54.5 ± 35	7.1 ± 6.3	19.2 ± 24.2
	Se	9.3 ± 6.6	7.6 ± 13	52.8 ± 24.4	29.5 ± 47.8	36.7 ± 10.4	17.6 ± 12.2	24.3 ± 22.9

Cu—copper, EAR—estimated average requirement, Se—selenium, Zn—zinc.

**Table 3:** The average content and median of TPC (mg GAE/100 g), DPPH (% Free Radical Scavenging), Cu (mg/kg), Se (µg/kg), and Cu (mg/kg) in the studied groups show significant statistical differences, taking into account the intended use of products for the age groups.

Type of Product (Sign)	Element	*n*	M ± SD	Min–Max	Me	Q1–Q3
For infant Aged 6–12 months (A)	TPC (mg GAE/100 g of fresh weight)	149	64.9 ± 59.7	0–234.6	42.6 *** B	21.1–98.7
	DPPH (% Free Radical Scavenging)		68.6 ± 31.9	0–99.6	79.9 *** C	56.8–93.3
	Cu (mg/kg of fresh weight)		7.5 ± 5.3	0.5–39.5	6.9 *** C	4.1–9.2
	Se (µg/kg of fresh weight)		54.8 ± 63.7	0–686.6	39.8 ** B, *** C	22.5–71.6
	Zn (mg/kg of fresh weight)		10.0 ± 14.6	0.6–93.3	3.7	2.2–12.0
For children between 1 and 3 years (B)	TPC (mg GAE/100 g of fresh weight)	62	33.4 ± 39.4	3.5–250.8	24.0 *** A	12.2–38.2
	DPPH (% Free Radical Scavenging)		73.9 ± 21.4	0–97.9	77.8 *** C	67.8–88.5
	Cu (mg/kg of fresh weight)		7.4 ± 3.2	2.8–20.4	6.9 *** C	5.3–8.6
	Se (µg/kg of fresh weight)		32.8 ± 23.9	5.2–136.9	26.1 ** B,*** C	17–41.7
	Zn (mg/kg of fresh weight)		11.1 ± 13.5	0.7–52.9	5.8	2.8–9.6
Without an age declaration (C)	TPC (mg GAE/100 g of fresh weight)	187	72.0 ± 82.5	0–525.4	50.8 *** B	15.9–99.1
	DPPH (% Free Radical Scavenging)		44.5 ± 44.9	0–91.7	53.4 *** A,B	30.1–76.9
	Cu (mg/kg of fresh weight)		12.2 ± 11.6	1.3–90.7	10.7 *** A,B	2.2–17.6
	Se (µg/kg of fresh weight)		118.1 ± 88.2	0–567.2	93.0 *** A,B	68.0–127.7
	Zn (mg/kg of fresh weight)		12.3 ± 14.1	0.1–69.4	5.5	2.2–17.6

** *p* < 0.01, *** *p* < 0.001. A— products for infants, 6–12 months, B—products for children between 1 and 3 years, C—products without an age declaration, Av—average, Cu—copper, DPPH—2,2-diphenyl-1-picrylhydrazyl radical scavenging test, GAE—gallic acid, M—mean, Max—maximum, Me—median, Min—minimum, *n*—number of samples, Q1—quartile 1, Q3—quartile 3, SD—standard deviation, Se—selenium, TPC—total phenolic content, Zn—zinc.

**Table 4:** The average content and median of TPC (mg GAE/100 g), DPPH (% Free Radical Scavenging), Cu (mg/kg), Se (µg/kg), and Cu (mg/kg) in the studied groups show significant statistical differences, taking into account the origin of the products.

Type of Product (Sign)	Elements	*n*	M ± SD	Min–Max	Me	Q1–Q3
Organic food (O)	TPC (mg GAE/100 g)	168	56.5 ± 63.5	0–507.2	34.3	16.8–78.2
	DPPH (% Free Radical Scavenging)		63.3 ± 31.7	0–98.3	72.7	48.6–85.5
	Cu (mg/kg)		9.3 ± 10.5	1.1–90.7	7.2	5.2–10.9
	Se (µg/kg)		84.2 ± 78.8	0–567.2	65.5	28.8–106
	Zn (mg/kg)		12.2 ± 13.9	0.5–93.3	6.8 ** C	2.8–16.3
Conventional food (C)	TPC	230	67 ± 71.3	0–525.4	44.6	15.8–96.8
	DPPH (% Free Radical Scavenging)		57.7 ± 43.2	0–99.6	69.5	43.8–89.5
	Cu (mg/kg)		10 ± 7.7	0.5–51.7	51.7	4.2–13.2
	Se (µg/kg)		76.9 ± 83.3	2.9–686.6	64.4	30.5–91.8
	Zn (mg/kg)		10.7 ± 14.4	0–75.7	4.3 ** C	2.0–12.4

** *p* < 0.01, C—conventional food, Cu—copper, DPPH—2,2-diphenyl-1-picrylhydrazyl radical scavenging test, GAE—gallic acid, M—mean, Max—maximum, Me—median, Min—minimum, *n*—number of samples, O—organic food, Q1—quartile 1, Q3—quartile 3, SD—standard deviation, Se—selenium, TPC—total phenolic content, Zn—zinc.

**Table 5:** Correlations between individual parameters (*p* < 0.05).

Type of Products	Parameter 1	Parameter 2	r	*p*
Dinners	TPC	Cu	−0.21	<0.05
	Zn	Cu	0.35	<0.001
	Zn	DPPH	0.23	<0.05
	DPPH	Cu	0.27	<0.005
Porridges	Se	Cu	0.29	<0.001
	Zn	TPC	−0.28	<0.001
	Cu	Zn	0.21	<0.001
	Cu	DPPH	0.38	<0.001
	DPPH	TPC	0.17	<0.005
Fruit and vegetable mousses	TPC	Zn	−0.32	<0.05
Baby drinks	TPC	Se	−0.28	<0.02
	Se	Zn	0.56	<0.001
	Se	Cu	0.38	<0.005
	Zn	Cu	0.27	<0.05
	Cu	DPPH	−0.32	<0.05
Dairy products	Se	Zn	0.45	<0.001
TOTAL	TPC	Se	0.22	<0.001
	TPC	Zn	−0.28	<0.001
	TPC	DPPH	0.17	<0.005
	Se	Cu	0.29	<0.001
	Cu	Zn	0.21	<0.001
	Cu	DPPH	0.38	<0.001

Cu—copper, DPPH—2,2-diphenyl-1-picrylhydrazyl radical scavenging test, Se—selenium, TPC—total phenolic content, Zn—zinc.

## Data Availability

Detailed data are available from the authors.

## Supplementary Materials

The following supporting information can be downloaded at: https://www.mdpi.com/article/10.3390/nu15143160/s1, Table S1: The content of TPC, DPPH, Cu, Se, Zn in tested dinners.; Table S2: The content of TPC, DPPH, Cu, Se, Zn in tested porridges.; Table S3: The content of TPC, DPPH, Cu, Se, Zn in tested mousses.; Table S4: The content of TPC, DPPH, Cu, Se, Zn in tested drinks.; Table S5: The content of TPC, DPPH, Cu, Se, Zn in tested hand-held snacks.; Table S6: The content of Cu, Se, Zn in tested dinners.
