# Supplementary material for: Evaluation of Selected Antioxidant Parameters in Ready-to-Eat Food for Infants and Young Children
Source: Nutrients. 2023 Jul 16;15(14):3160. doi: 10.3390/nu15143160 (PMC10385023; doi:10.3390/nu15143160)
Supplement: Supplementary file 1 [file nutrients-15-03160-s001.zip › nutrients-2502431-SI.pdf]

nutrients MDPI

Supplementary materials

Evaluation of Selected Antioxidant Parameters in Ready-to-eat Food For Infants And Young Children

Anita Żmudzińska*, Anna Puścion-Jakubik, Jolanta Soroczyńska, Katarzyna Socha

Department of Bromatology, Faculty of Pharmacy with the Division of Laboratory Medicine, Medical University of Bialystok, Mickiewicza 2D Street, 15-222 Bialystok, Poland; anita.zmudzinska@sd.umb.edu.pl (A.Ż); anna.puscion-jakubik@umb.edu.pl (A.P.-J.); jolanta.soroczynska@umb.edu.pl (J.S.); katarzyna.socha@umb.edu.pl (K.S.)

* Correspondence: anita.zmudzinska@sd.umb.edu.pl; Tel.: +48-8574-854-69.

Table S1. The content of TPC, DPPH, Cu, Se, Zn in tested dinners.

Type of Dinners		TPC (mg GAE/100 g)	DPPH (% Free Radical Scavenging)	Cu (mg/kg)	Se (µg/kg)	Zn (mg/kg)	Cu:Zn molar ratio
**Poultry**		28.1 ± 17.3	68.4 ± 24.2	7.3 ± 2.6	18.9 ± 8.3	5.4 ± 4	1.9 ± 1.1
**dinners**		(0-74.5)	(0-93.1)	(3.1-15.6)	(5.3-39.5)	(1.4-16.4)	(0.4-4.8)
**(*n* = 23)**		25.6 (15.5-38.5)	77.4 (62.5-82.7)	7.1 (62.5-82.7)	16.6 (13.2-25.5)	3.6 (3.1-6.7)	1.6 (1.3-2.4)
**Beef**		36.5 ± 17.5	65.5 ± 18.9	7.7 ± 2.2	23.4 ± 11.4	6.4 ± 1.9	1.5 ± 1.1
**dinners**		(7.3-80.5)	(19.7-91.8)	(4.5-11.6)	(8.3-44.9)	(1.8-8.5)	(0.5-5.4)
**(*n* = 16)**		35.8 (25.9-46.2)	69.6 (55.1-78.1)	7.5 (5.8-9.4)	21.2 (15.7-27.2)	6.7 (5.5-7.9)	1.1 (0.9-1.8)
**Pork**		23.3 ± 16.7	66.5 ± 27.8	7.1 ± 1.5	28.5 ± 16.9	3.7 ± 1.3	2.2 ± 0.8
**dinners**	Av ± SD	(4.7-54.8)	(0-96.9)	(4-10.4)	(5.3-76.4)	(1.5-6.5)	(1.3-4.0)
**(*n* = 15)**	Min-Max	19.9 (9.7-33.6)	70.2 (63.6-84.5)	7.2 (6.3-7.9)	24.9 (17-36.8)	3.8 (3.3-4.1)	1.8 (1.7-2.5)
**Fish**	Me	22.9 ± 17.6	62.2 ± 19.3	7.7 ± 2.9	26.8 ± 16.6	3.7 ± 2.3	2.7 ± 1.5
**dinners**	Q1-Q3	(2.4-61.3)	(25.9-91.9)	(3.7-12.6)	(7-72)	(1.3-9.6)	(0.8-6.4)
**(*n* = 19)**		17.3 (7.6-33.3)	64.8 (48.6-80.6)	7.5 (5.1-10.1)	21.7 (15.1-32.2)	17.3 (7.6-33.3)	2.5 (1.4-3.3)
**Rabbit**		30.2 ± 26.4	48.5 ± 35.4	6 ± 3.3	28 ± 16.7	4 ± 4.2	1.9 ± 0.8
**dinners**		(1.1-84.4)	(0-91)	(1.9-11.2)	(6.7-60.2)	(0.8-16.2)	(0.7-3.2)
**(*n* = 11)**		30.2 (5.6-41.2)	55.4 (14.5-82.4)	7.1 (1.9-8.6)	22.8 (16.4-39.8)	2.7 (1.9-4.2)	2.1 (1.0-2.4)
**Vegetarian**		34.3 ± 37.2	40.5 ± 40.1	7.4 ± 2.5	36.7 ± 11.4	7.8 ± 9.6	1.5 ± 0.8
**dinners**		(3.3-181.6)	(0-87.7)	(3.4-15.5)	(20.5-58.8)	(2.3-46.3)	(0.1-3.0)
**(*n* = 19)**		26.6 (20.7-35.7)	60.4 (14.1-66.7)	7 (6.2-8)	31.9 (26.9-47.7)	4.7 (3.9-7.8)	1.3 (0.8-2.1)

Av—average, Cu—copper, DPPH—2,2-diphenyl-1-picrylhydrazyl radical scavenging test, GAE—gallic acid, Max—maximum, Me—median, Min—minimum, n—number of samples, Q1—quartile 1, Q2— quartile 2, SD—standard deviation, Se—selenium, TPC—total phenolic content, Zn—zinc

Table S2. The content of TPC, DPPH, Cu, Se, Zn and Cu:Zn molar ratio in tested porridges.

Type of Porridges		TPC (mg GAE/100 g)	DPPH (% Free Radical Scavenging)	Cu (mg/kg)	Se (µg/kg)	Zn (mg/kg)	Cu:Zn molar ratio
**Milk porridges (*n* = 12)**	Av ± SD<br>Min-Max<br>Me Q1-Q3	52.7 ± 54.9<br>(3.6-205.8)<br>33.9 (16.6-71.4)	74.2 ± 21<br>(44-97)<br>93.3 (49.4-90.7)	5.6 ± 2.1<br>(3-9.2)<br>5.6 (3.6-7.2)	32.2 ± 25.8<br>(9.5-106.2)<br>27.4 (16.9-34.5)	36.9 ± 22.8<br>(12.4-93.3)<br>34.4 (18.5-44)	0.2 ±0.1<br>(0.1-0.4)<br>0.2 (0.1-0.2)
**Milk and fruit porridges (*n* = 14)**		44.9 ± 39.7<br>(7.5-138.2)<br>39.3 (15.4-51)	61.1 ± 30.8<br>(0-92.7)<br>68.3 (59.1-79.9)	2.2 ± 1.2<br>(1.1-4.9)<br>2 (59-80)	109 ± 169.6<br>(11.2-686.6)<br>70.4 (56.7-74.6)	23.8 ± 9.0<br>(12-38.5)<br>22.7 (18.2-28.8)	0.1 ± 0.1<br>(0-0.2)<br>0.1 (0.1-0.2)
**Cereal gluten porridges (*n* = 10)**		34.7 ± 31.2<br>(5.5-88.4)<br>26.2 (6-69.2)	57.1 ± 37<br>(0-84.4)<br>72.7 (54.7-79.8)	3.9 ± 2.2<br>(0.5-7.8)<br>3.8 (2.5-5.4)	32.2 ± 18<br>(5.2-70.6)<br>32.2 (23.7-40.1)	27.8 ± 14.9<br>(3.7-52.9)<br>25 (18.4-39.3)	0.2 ± 0.1<br>(0-0.3)<br>0.2 (0.1-0.2)
**Cereal gluten free porridges (*n* = 14)**		45.4 ± 54.9<br>(4.4-206)<br>25.6 (12.5-59.5)	79.2 ± 12.4<br>(44-90.3)<br>83.7 (75.4-87.4)	3.7 ± 1.9<br>(1-7.5)<br>3.7 (75.4-87.4)	39 ± 23.7<br>(17.2-94.3)<br>32 (19.8-54.2)	33.4 ± 15.8<br>(9.4-75.7)<br>31.5 (26.4-36.9)	0.1 ± 0.1<br>(0-0.4)<br>0.1 (0.1-0.2)

Av—average, Cu—copper, DPPH—2,2-diphenyl-1-picrylhydrazyl radical scavenging test, GAE—gallic acid, Max—maximum, Me—median, Min—minimum, n—number of samples, Q1—quartile 1, Q2— quartile 2, SD—standard deviation, Se—selenium, TPC—total phenolic content, Zn—zinc

Table S3. The content of TPC, DPPH, Cu, Se, Zn and Cu:Zn molar ratio in tested mousses.

Type of Mousses		TPC (mg GAE/100 g)	DPPH (% Free Radical Scavenging)	Cu (mg/kg)	Se (µg/kg)	Zn (mg/kg)	Cu:Zn molar ratio
**Fruit and vegetable (*n* =48)**	Av ± SD<br>Min-Max<br>Me Q1-Q3	118.1 ± 64.5<br>(6.5-250.8)<br>114.2 (65.8-167.4)	93 ± 6.7<br>(64.7-99.6)<br>95.3 (92.3-97)	11.6 ± 7.1<br>(2.2-39.5)<br>9.6 (7.7-14.2)	78.1 ± 34.9<br>(24.1-148.6)<br>81 (65.9-84.3)	2.3 ± 1<br>(0.7-4.8)<br>14.2 (65.8-167.4)	5.4 ± 3.8<br>(3.0-12.7)<br>3.5 (3.1-4.6)
**Fruit (*n* = 14)**		83.1 ± 64.9<br>(9-187.6)<br>76.4 (22-122.5)	81.1 ± 39.8<br>(0-99.5)<br>95,9 (87.2-97.4)	8.3 ± 3.7<br>(4-16.4)<br>7.7 (6.3-9.9)	76.2 ± 34.8<br>(0-150.1)<br>73 (53.4-100.2)	1.7 ± 64.9<br>(9-187.6)<br>1.6 (1.1-2.3)	7.7 ± 6.1<br>(1.2-30.9)<br>5.6 (4.2-9.9)
**Fruit and cereal (*n* = 6)**		92.5 ± 62.3<br>(29.7-199.8)<br>87.1 (36.4-115)	95.6 ± 1.43<br>(26.7-93.3)<br>95.6 (94.8-96.6)	11.6 ± 7.4<br>(3-23.9)<br>9.2 (8-16.4)	91.3 ± 48.9<br>(37.7-175.7)<br>86.9 (53.3-107.2)	2.1 ± 0.7<br>(3.2-1.8)<br>2 (8-16.4)	5.6 ± 2.8<br>(1.5-10.2)<br>5.2 (4.8-6.9)
**Fruit and dairy (*n* = 6)**		47.7 ± 39.4<br>(16.4-119.3)<br>36.7 (19.2-57)	93.8 ± 5.1<br>(86.1-99.6)<br>95.3 (89.3-97)	12 ± 13.8<br>(2.6-39.5)<br>7.8 (4-10.4)	78 ± 42<br>(38.8-145)<br>70.7 (45.8-95.9)	3 ± 0.8<br>(2.2-4.3)<br>2.8 (2.3-3.4)	4.7 ± 6.2<br>(0.8-17.2)<br>2.5 (1.9-3.2)
**Vegetables (*n* =4)**		31.7 ± 18.6<br>(9-50.6)<br>33.6 (16.9-46.6)	55.4 ± 57.6<br>(0-87.2)<br>82.7 (1.2-3.1)	7.1 ± 1.2<br>(2.2-11.1)<br>7.7 (4.8-9.5)	106.7 ± 39.5<br>(54.1-149.9)<br>111.5 (82.1-131.3)	2.1 ± 1.2<br>(1-3.7)<br>2 (1.2-3.1)	3.7 ± 1.7<br>(2.0-5.6)<br>3.5 (2.3-5.1)

Av—average, Cu—copper, DPPH—2,2-diphenyl-1-picrylhydrazyl radical scavenging test, GAE—gallic acid, Max—maximum, Me—median, Min—minimum, n—number of samples, Q1—quartile 1, Q2— quartile 2, SD—standard deviation, Se—selenium, TPC—total phenolic content, Zn—zinc

Table S4. The content of TPC, DPPH, Cu, Se, Zn and Cu:Zn molar ratio in tested drinks.

Type of Drinks		TPC (mg GAE/100 g)	DPPH (% Free Radical Scavenging)	Cu (mg/kg)	Se (µg/kg)	Zn (mg/kg)	Cu:Zn molar ratio
**Fruit drinks and Water (*n* = 21)**	Av ± SD	43.1 ± 47	18.9 ± 36.8	4.7 ± 2.4	195.2 ± 160.3	10.0 ± 11.8	6.0 ± 14.5
	Min-Max	(0-164)	(0-76.8)	(1.6-8.1)	(28.8-567.2)	(0.1-39)	(0.2-62.5)
	Me Q1-Q3	32.2 (5.3-70.1)	35.8 (0-45.1)	5.5 (1.9-6.6)	105.7 (82.5-330.3)	3.2 (1.1-18)	6.0 (0.4-62.5)
**Fruit juices (*n* = 43)**	Av ± SD	91.9 ± 53	24.7 ± 50.1	2.7 ± 1.5	78.4 ± 36.6	1.4 ± 1.1	2.9 ± 2.0
	Min-Max	(0-241.9)	(0-87.4)	(1.3-8.7)	(0-201.8)	(0.4-5.5)	(0.3-10.5)
	Me Q1-Q3	86.9 (57.7-117.3)	33.7 (4.4-56.6)	1.9 (1.7-4)	77.5 (47.2-102.7)	1 (0.7-1.9)	2.6 (1.6-4.0)

Av—average, Cu—copper, DPPH—2,2-diphenyl-1-picrylhydrazyl radical scavenging test, GAE—gallic acid, Max—maximum, Me—median, Min—minimum, n—number of samples, Q1—quartile 1, Q2— quartile 2, SD—standard deviation, Se—selenium, TPC—total phenolic content, Zn—zinc

Table S5. The content of TPC, DPPH, Cu, Se, Zn and Cu:Zn molar ratio in tested snacks "for the hand".

Type of Snacks "for the hand"		TPC (mg GAE/100 g)	DPPH (% Free Radical Scavenging)	Cu (mg/kg)	Se (µg/kg)	Zn (mg/kg)	Cu:Zn molar ratio
**Waffle / Crips (*n* = 29)**	Av ± SD	80.8 ± 110.6	67.9 ± 29.8	10.8 ± 3.6	80.6 ± 21	15.5 ± 8.4	1.0 ± 0.7
	Min-Max	(0-507.2)	(0-89.7)	(2.9-20.6)	(30.1-110.9)	(3.9-41.5)	(0.2-2.9)
	Me Q1-Q3	42 (12.1-94)	76.9 (70.6-81.8)	10.7 (8.7-12.4)	83.4 (69.5-95.8)	14.3 (11.4-19.2)	1.0 (0.5-1.1)
**Biscuits / Cookies (*n* = 22)**	Av ± SD	61.3 ± 109.7	63.5 ± 39.2	18.8 ± 23.6	69.3 ± 19.5	16.4 ± 13.6	1.3 ± 1.2
	Min-Max	(7.2-525.4)	(0-91.7)	(3.8-90.7)	(24.1-97.8)	(5.5-69.4)	(0.3-6.2)
	Me Q1-Q3	24.1 (16.6-65.4)	80.6 (48.3-86.1)	11.9 (8.4-15.6)	72.4 (61.1-81.6)	14 (8.2-15.8)	1.3 (0.7-1.3)
**Fruit bars (*n* = 12)**	Av ± SD	49.7 ± 69.8	69.2 ± 15.3	14.5 ± 6.7	80.2 ± 25.1	20.1 ± 9.8	0.9 ± 0.6
	Min-Max	(0-254.8)	(45.1-89.8)	(7.6-29.1)	(36.6-122.2)	(7.8-38.5)	(0.3-1.8)
	Me Q1-Q3	22.7 (15.6-51.8)	22.7 (15.6-51.8)	12 (10.9-15.8)	77.9 (64.3-99.4)	20.6 (12.5-24.6)	0.9 (0.4-1.5)

Av—average, Cu—copper, DPPH—2,2-diphenyl-1-picrylhydrazyl radical scavenging test, GAE—gallic acid, Max—maximum, Me—median, Min—minimum, n—number of samples, Q1—quartile 1, Q2— quartile 2, SD—standard deviation, Se—selenium, TPC—total phenolic content, Zn—zinc

Table S6. The content of Cu, Se, Zn and Cu:Zn molar ratio in tested dinners.

Type of Dairy		Cu (mg/kg)	Se (µg/kg)	Zn (mg/kg)	Cu: Zn molar ratio
**Yellow cheese (*n* = 28)**	Av ± SD	17.9 ± 7.4	183. ± 99.3	38.4 ± 11.6	0.9 ± 1.1
	Min-Max	(11.5-38.6)	(48.1-454.7)	(4.4-54.4)	(0.3-4.1)
	Me Q1-Q3	15.3 (13.3-18)	164.2 (120.2-214.5)	40.5 (37.2-42.7)	0.9 (0.3-0.7)
**Yogurt (*n* = 32)**	Av ± SD	23.8 ± 7.6	147 ± 68.2	4.4 ± 1.4	6.4 ± 2.4
	Min-Max	(13.3-51.7)	(47.5-341.3)	(2-7.4)	(1.8-11.2)
	Me Q1-Q3	22.1 (20.4-26.6)	132.6 (110.4-174.5)	1.6 (3.3-5.1)	5.8 (5.0-8.1)

Av—average, Cu—copper, GAE—gallic acid, Max—maximum, Me—median, Min—minimum, n—number of samples, Q1—quartile 1, Q2— quartile 2, SD—standard deviation, Se—selenium, Zn—zinc
